# Knowledge and smart health device use in racial-ethnic minority older adults with hypertension: a qualitative study

**DOI:** 10.1093/geroni/igag083

**Published:** 2026-07-20

**Authors:** Jany Sun, Yanjun Dong, Jimmie Boliboun, Katherine Koo, Michael Cui, Victoria M Rizzo, Jeannine M Rowe

**Affiliations:** Department of Internal Medicine, Rush University Medical Center, Chicago, Illinois, United States; School of Social Welfare, College of Integrated Health Sciences, University at Albany, State University of New York, Albany, New York, United States; Department of Internal Medicine, Rush University Medical Center, Chicago, Illinois, United States; Department of Family and Preventive Medicine, Rush University Medical Center, Chicago, Illinois, United States; Department of Internal Medicine, Rush University Medical Center, Chicago, Illinois, United States; School of Social Welfare, College of Integrated Health Sciences, University at Albany, State University of New York, Albany, New York, United States; Department of Social Work, College of Letters and Science, University of Wisconsin-Whitewater, Whitewater, Wisconsin, United States

**Keywords:** Smart health devices, Digital divide, Remote patient monitoring, Critical race theory, Andersen’s behavioral model

## Abstract

**Background and Objective:**

Smart health devices (SHDs) can improve hypertension management. Yet, SHD use among racial-ethnic minority older adults remains low, delaying potential benefits and exacerbating health disparities. Knowledge is a key determinant of SHD adoption; however, its influence on SHD use is not well understood. This study examines how knowledge affects SHD use among racial-ethnic minority older adults with hypertension.

**Research Design and Methods:**

We conducted an exploratory qualitative study using semi-structured interviews with 20 racial-ethnic minority older patients aged 60+ with hypertension and prescribed a SHD. Thematic and classical content analysis were used to analyze the interview data.

**Results:**

Multiple dimensions of knowledge, including prior awareness, sources of information, and gaps in knowledge, were identified as factors in using SHDs. Sixty percent had no knowledge of SHDs prior to being prescribed one. All received education about how to use the SHD from their providers, with many receiving support from others. Gaps in understanding technology, such as how SHDs transmit information, the security of information, and the potential health benefits, were observed. When knowledge gaps were addressed, it resulted in increased SHD utilization as well as improved health behaviors and emotional impacts.

**Discussion and Implications:**

Through integrating Critical Race Theory and Andersen’s model, this study demonstrates that limited baseline awareness of SHD among racial-ethnic minority older adults is shaped by structural inequities. Provider education and support facilitated SHD engagement. Culturally responsive education, ongoing clinical support, and community-based education are essential to improving SHD utilization and self-monitoring behavior.

Innovation and Translational Significance: Racial-ethnic minority older adults face structural inequities in smart health device (SHD) adoption, widening cardiovascular disease disparities. Integrating Critical Race Theory and Andersen’s Behavioral Model, this exploratory qualitative study used semi-structured interviews with 20 racial-ethnic minority older adults to examine how knowledge influences SHD utilization. Findings show that while 60% lacked SHD awareness, guidance from providers and ongoing clinical support improved device engagement. These insights guide the design of culturally responsive clinical workflows and community-based digital education programs, while advocating equitable health policies to eliminate digital disparities and improve health outcomes.

## Background and objectives

Hypertension is a primary risk factor for cardiovascular (CV) disease, a leading cause of death in the United States (Centers for Disease Control and Prevention, 2023a). Nearly half of adults have hypertension, but only one in four achieve blood pressure control (Centers for Disease Control and Prevention, 2023b). The economic burden exceeds $100 billion annually, with individuals facing nearly $2,000 higher annual healthcare costs compared to peers without hypertension (Centers for Disease Control and Prevention, 2023b; Yang et al., 2024).

Adults aged 65 and older experience disproportionately high rates of CV morbidity and mortality, with prevalence approaching 70% (Glazier, 2022). These disparities are further exacerbated among racial-ethnic minority older adults, with hypertension prevalence rates of 58% among Black Americans and 43.7% among Hispanic/Latino populations (Shan et al., 2020). Despite evidence-based guidelines, hypertension remains largely uncontrolled in these groups due to socioeconomic challenges, patient-related barriers, and healthcare system limitations (Aggarwal et al., 2021; Heaton et al., 2024; Kulkarni et al., n.d.; Muntner et al., 2020).

From a Critical Race Theory (CRT) perspective, these disparities reflect structural racism embedded within social and healthcare systems rather than solely individual factors (Ford & Airhihenbuwa, 2010). Structural inequities—such as residential segregation, limited access to healthy foods, and reduced access to high-quality care contribute to both the development and poor control of hypertension. Additionally, experiences of discrimination, medical mistrust, and exclusion from decision-making can reduce engagement in care and adherence to treatment (Freeman et al., 2017). CRT highlights the routine nature of racism, emphasizing how cumulative barriers shape health and outcomes (Ford & Airhihenbuwa, 2010).

Smart health devices (SHD), particularly smart blood pressure monitors, can improve hypertension control among high-risk populations (Shan et al., 2020). SHDs wirelessly transmit blood pressure readings to electronic health record systems and clinical applications for data recording and analysis (Ciemins et al., 2018). This self-monitoring approach enhances patient self-efficacy and supports provider-guided interventions, such as patient education and medication adjustments (Hellem et al., 2024; Stergiou et al., 2018). Studies document that SHD utilization significantly reduced in-office blood pressure readings and increased attainment of targeted blood pressure measures, as well as improved long-term hypertension control (Chandrasekaran et al., 2021; Jo et al., 2019).

Despite their potential, SHDs remain underutilized among racial-ethnic minority older adults. From a CRT perspective, these disparities are not solely the result of individual-level factors but are rooted in broader structural inequalities that shape access to resources and healthcare engagement (Ford & Airhihenbuwa, 2010). Structural racism—manifested through unequal access to broadband infrastructure, income disparities, and underinvestment in racial-ethnic minority communities systematically limits opportunities for racial-ethnic minority older adults to adopt digital technologies. One key mechanism through which these structural inequities operate is the digital divide in internet access and quality.

These structural inequities are reflected in persistent disparities in internet access and quality. Inconsistent connectivity directly influences racial-ethnic minority older adults’ decisions to use SHDs. For example, Li and colleagues found that only 66% of Black American households and 61% of Hispanic/Latino households had internet access, approximately 10% to 15% lower than White and Asian households (Y. Li et al., 2023). In addition, internet speeds are significantly different in racial-ethnic minority households (Rodriguez-Elliott & Vachuska, 2023), further limiting the effective use of digital health tools. Although smartphone ownership has increased in recent years, many individuals cannot afford the data services required to transmit SHD data to clinicians (Harmon Still et al., 2018). As a result, limited and lower-quality connectivity restricts access to electronic health records and online health information.

Beyond issues of access, interpersonal and perceptual factors further shape SHD utilization. Racial-ethnic minority older adults often report a preference for in-person clinician communication and express concerns about confidentiality and data security. In a study conducted by Ladin and colleagues, participants described dissatisfaction with the lack of interpersonal interaction in digital health innovations, fearing that clinicians may not understand their concerns and expressing skepticism about how data are managed (Ladin et al., 2021). These concerns, when considered alongside structural barriers, contribute to lower adoption of SHDs. Consequently, the stark differences in SHD use between racial-ethnic minority and White older adults further widen existing healthcare disparities.

From a CRT perspective, knowledge of SHD is not distributed equally across populations. It is shaped by structural conditions that influence access to information and digital resources. Structural racism and institutional barriers contribute to disparities in education, technology access, and digital literacy development, limiting awareness and adoption of SHD among racial-ethnic minority older adults. In addition, historical experiences of discrimination contribute to a lower level of trust in the healthcare system among racial-ethnic minority communities. Previous studies have shown that medical mistrust is linked to reduced adherence to treatment recommendations, lower satisfaction with care, and poor health outcomes (Bazargan et al., 2021). Medical mistrust renders racial-ethnic minority older adults reluctant to adopt and engage with SHD, widening existing knowledge gaps of SHD. Consequently, knowledge gaps of SHD reflect broader structural inequities rather than individual deficits. Understanding how racial-ethnic minority older adults acquire and apply knowledge of SHD is critical for identifying the mechanisms by which structural inequities influence digital health technology utilization and perpetuate health disparities.

Beyond these structural and interpersonal barriers, gaps in knowledge play a pivotal role in SHD use among older adults. Norman and Skinner’s concept of eHealth knowledge suggests that knowledge is multidimensional, with the need to know how to use the device, as well as comprehend, evaluate, and apply health-related benefits (Norman & Skinner, 2006). A Pew Research Center study found that one-third of older adults are uncomfortable performing basic digital tasks, such as using SHDs (Anderson & Perrin, 2017). Less is known about the multiple dimensions of knowledge and the effect on SHD utilization, particularly among racial-ethnic minority older patients. Lack of knowledge contributes to passive engagement with healthcare providers, disengagement from proactive health monitoring, and negative health outcomes (Aaby et al., 2017). Exacerbating these barriers is the limited time clinicians have with patients in the clinical settings. Understanding how knowledge influences SHD is critical.

### Theoretical framework

Andersen’s Expanded Behavioral Model of Health Services Use provides a multidimensional framework for understanding health behaviors through predisposing, enabling, and need factors, with knowledge identified as a key psychosocial determinant influencing service utilization (Travers et al., 2020). This model incorporates race and ethnicity as structural and environmental factors that shape access to resources and healthcare, offering a nuanced understanding of health behaviors among diverse populations.

Within this framework, enabling factors such as broadband access, affordability, and digital infrastructure reflect structural barriers contributing to the digital divide, while psychosocial factors—particularly knowledge—are shaped by unequal access to information and digital literacy. Knowledge, along with attitudes, social norms, and perceived control, plays a central role in decision-making regarding SHD use.

Accordingly, this study uses Andersen’s model to examine the role of knowledge as a key mechanism through which the digital divide and structural inequities influence (Bradley et al., 2002) SHD use among racial-ethnic minority older adults. By integrating CRT with Andersen’s model, we move beyond individual-level explanations to consider how systemic barriers shape both access to knowledge and capacity to act on that knowledge. This approach allows for a more comprehensive understanding of disparities in digital health technology use and underscores the importance of addressing both knowledge gaps and structural conditions that contribute to promoting equitable adoption of SHDs.

## Research design and method

### Study design

We conducted an exploratory qualitative study using semi-structured interviews to gain an in-depth understanding of SHD use among racial-ethnic minority older adults. This approach is well-suited for examining under-researched phenomena (Green & Thorogood, 2018). The interdisciplinary research team included two PhD researchers with expertise in healthcare research and qualitative methods, a physician with training in informatics and expertise in clinical CV and hypertension care, a medical student with clinical training in CV and hypertension management, a PhD student specializing in qualitative analysis, and two research assistants with experience in healthcare settings. Ethics approval for the study was received from Rush University Medical Center’s (RUSH) institutional review board (IRB). Study findings are reported in accordance with the Consolidated Criteria for Reporting Qualitative Research (COREQ) (Tong et al., 2007).

### Sample recruitment

We purposely sampled racial-ethnic minority patients from a larger study being conducted at RUSH that focused on health equity. The goal of the larger study was to reduce cardiovascular risk factors and address social barriers faced by marginalized communities through an interdisciplinary, team-based remote monitoring program. Patients in the larger study were adults aged 18 and older who had a diagnosis of hypertension. As part of the larger study, patients received the HealthSnap Blood Pressure Monitor, a smart blood pressure monitor, to manage their hypertension, and education, which was provided by a pharmacist, on how to use the monitor. The larger study included 105 participants. Among these participants, 94 (90%) were Black Americans, and 11(10%) were Hispanic/Latino. None were other minority groups.

We targeted Black Americans and Hispanic/Latino patients aged 60 and older for recruitment into the current study. To enhance diversity among these groups, we stratified groups by gender and age cohorts of young-old (ages 60–74), middle-old (ages 75–84), and oldest-old (aged 85 and older). Our goal was to recruit and interview four Black males and four Black females from each age cohort, as well as one Hispanic/Latino male and one Hispanic/Latino female from each age cohort. Eligibility criteria included being aged 60 or older, cognitively intact, and able to read and understand English. Additionally, consistent with the eligibility of the larger study, patients had a diagnosis of hypertension and were prescribed a smart blood pressure monitor. We identified 49 patients who met the eligibility criteria—46 were Black American (93.9%), and 3 were Hispanic (6.1%).

Recruitment was conducted by telephone. The first author (JS), who was not a part of the larger study nor had previous involvement with eligible patients (*n *= 49), called and invited them to participate. Twenty patients were not reached (40.8%), nine declined to participate (18.4%), and 20 (40.8%) demonstrated an interest in the study (38.8%). The 20 patients who expressed interest voluntarily agreed to participate, provided written informed consent, and were enrolled in the current study.

### Data collection

One-to-one semi-structured interviews were also conducted by the first author via Webex, which is a web-based video and telephone platform, between December 2023 to July 2024. Participants had the option to complete the interview using either video or telephone. Interviews were audio recorded and converted into text files, which served as the data for analysis.

The semi-structured interview guide (Table 1) developed by the primary investigator (JMR) and co-investigator (VMR) was informed by Andersen’s model and the digital literacy framework (Bradley et al., 2002; Norman & Skinner, 2006; Travers et al., 2020). The guide assessed participants’ prior knowledge of SHDs, their use and purpose of SHDs, and factors influencing future SDH use. Expert feedback from two additional team members (MC and JS) led to minor revisions and the finalization of the guide. The first author followed the order of the interview guide, unless the participant answered a question without being asked.

**Table 1:** Interview guide.

Target	Questions	Prompts/probes
**(A) All participants**	Prior to being prescribed the smart blood pressure cuff by [doctor name], had you heard of this/these type of device(s)?	If yes ○ Where? ○ From whom?
	2) Are you currently using the blood pressure cuff as prescribed by [doctor name]?	If yes ○ What is the main reason(s) you decided to accept and use the smart blood pressure cuff? ○ Did someone influence your decision to accept or use the smart blood pressure cuff? Who is the person(s)? If no ○ What is the main reason(s) you decided not to accept or not to use the smart blood pressure cuff? ○ Did someone influence your decision to not accept or not to use the smart blood pressure cuff? Who is the person(s)?
	3) How often did [doctor name] recommend you use the smart blood pressure cuff to check your blood pressure?	Are you checking it at the recommended frequency? Why or why not?
	4) How often were you asked by [doctor name] to send your blood pressure readings to your them or the healthcare team?	Are you sending the information as requested? Why or why not?
**(B) Participants who answered *yes* to question #2 in (A)**	In what ways has using the smart blood pressure cuff changed your life?	How? What else? Please provide some examples.
	2) Are you or have you experienced any challenges using your smart blood pressure cuff?	What are/were some of the challenges? Please be specific. These can be things like simply using the device, sending the data to your doctor or healthcare team, or not having cellular data or internet to send the information.
	3) Is there someone who helps you use your smart blood pressure cuff?	If yes ○ Who is the person(s)? ○ What kind of help do they give you? If no ○ Do you wish you had someone to help you use the smart blood pressure cuff? If yes, who do you wish would help you?
	4) What would you need to continue using your smart blood pressure cuff?	Are there specific things that could get in the way of using the smart blood pressure cuff? What else?
**(C) Participants who answered *no* to question #2 in (A)**	What are some reasons that you are not using your smart blood pressure cuff?	Are there specific things that stand in the way of using the smart blood pressure cuff? ○ What are these things? ○ What else?
	2) What if you could get the help/support with [refer to reasons reported in previous response], would you consider using the smart blood pressure cuff?	What would make you want to use the smart blood pressure cuff? Why?
	3) How do you think using a smart blood pressure cuff might change your life?	Would it impact your health? Your feelings about your health? Your family’s feelings about your health?
**(D) All participants**	What else would you like to tell me about your smart blood pressure cuff that I did not ask?	This can be anything.

All 20 enrolled patients completed the interviews. Interviews lasted an average of 28 minutes (range: 20–45 minutes), yielding rich, in-depth data for analysis (Malterud et al., 2016). Participants received a $50 electronic gift card upon completion of the interview as a token of appreciation. Transcripts generated by WebEx were verified for accuracy and cleaned by two project assistants (JB and KK) and the first author (JS). Demographic data were obtained from each patient’s electronic health record. These data were analyzed and managed using SPSS 28.

### Data analysis

Transcripts were imported into ATLAS.ti, version 24.1.1 for analysis using thematic analysis (Naeem et al., 2023). The analysis combined inductive and deductive coding approaches (Kaur et al., 2025). Peer debriefing was used to enhance methodological rigor (Nowell et al., 2017). Initially, two researchers (YD and JMR) independently read the transcripts and inductively identified and coded concepts that flowed freely from the data. One researcher (YD) then mapped the free-floating codes to the psychosocial dimension of Andersen’s model—specifically the *knowledge* domain using a direct analysis approach (deductive). Both researchers met to review and refine this mapping through iterative discussion until agreement was reached. Second, the researcher (YD) extracted codes related to *knowledge* and developed preliminary themes. These themes were discussed collaboratively with the second researcher (JMR), with consensus achieved without further iteration. Finally, to enhance the validity of our findings, we employed classical content analysis procedures that involved quantifying codes.

### Artificial intelligence tools used

ChatGPT (OpenAI) was used to assist with language editing and flow in the discussion section. All content was critically reviewed and revised by the authors, who take full responsibility for the final manuscript.

## Results

This study explored the experiences of 20 racial-ethnic minority older adults using a smart blood pressure monitor, focusing on how knowledge influenced their use of this type of SHD. All participants had chronic conditions, such as hypertension or CV disease, and the smart blood pressure monitor was integrated into their healthcare plans as a management tool. The average age of participants was 70.7 years (*M *= 70.7, *SD *= 10.3), with most identifying as Black American (*n *= 19, 95%) and female (*n *= 11, 55%) (Table 2).

**Table 2:** Demographic information of study participants (*N *= 20).

Characteristics	Response	*n*	%
**Race/ethnicity**	Black American	19	95
	Hispanic/Latino	1	5
**Gender**	Male	9	45
	Female	11	55
**Age**	60–70	13	65
	70–80	3	15
	80–90	2	10
	90 and over	2	10
**Education**	Middle school	1	5
	Some high school	3	15
	High school diploma	4	20
	Some college	2	10
	College degree	6	30
	Missing	4	20
**Employment**	Employed	2	10
	Full time	5	25
	Retired	7	35
	Unemployed	6	30
**Sexual orientation**	Straight	14	70
	Missing	6	30
**Marital status**	Single	7	35
	Married	3	15
	Domestic partner	1	5
	Divorced	4	20
	Widowed	4	20
	Other	1	5
**Insurance**	Medicaid	6	30
	Medicare	3	15
	Private	4	20
	Medicaid and Medicare	6	30
	Medicare and private	1	5
**Chronic health Condition**	Hypertension	20	100
	Diabetes	15	75
	Hypercholesterolemia	5	25
	Vascular diseases	11	55
	Others	10	5

Knowledge of smart health devices emerged as a pivotal factor influencing the use of SHDs and the integration of these devices into daily routines. We identified five thematic categories of knowledge related to SHD use: baseline awareness, information sources, knowledge gaps, facilitators of engagement, and behavioral impacts (Table 3).

**Table 3:** Quantification of factors, domains, themes, and dimensions through classical analysis (*N *= 20).

Category	*N*
**Factors**	20
Psychosocial	20
**Domains**	
Knowledge	20
**Major themes and their dimensions**	
Baseline awareness	12
Sources of information	
*Healthcare provider*	20
*Family members/friends*	4
*Self-learning*	3
Knowledge gaps	12
Facilitators of engagement	
*Healthcare provider*	2
*Understanding & guidance*	18
Behavioral impacts	17

*Note.* “*N*” represents the number of times each code was observed across participant interviews. A higher count reflects more frequent identification of the theme during the analysis.

### Baseline awareness

Twelve of 20 participants (60%) reported a lack of awareness of SHDs before being prescribed one. This lack of familiarity was evident in responses such as “No” (Interviewee 9) and “No, I’ve never heard of it before” (Interviewee 8). Some participants illustrated the device’s novelty in their healthcare experience. For example, one participant remarked, “No. Oh, you mean, like a blood pressure cuff that then transmits data electronically? No, I wasn’t familiar with that kind of technology” (Interviewee 5).

This widespread lack of awareness highlights a significant gap in the dissemination of information about SHDs to racial-ethnic minority older adults. Many participants encountered these devices reactively, only after being prescribed them by a healthcare provider, rather than through proactive education or broader public health campaigns. This reactive introduction may have limited participants’ initial trust and comfort with the technology, potentially delaying its use. Given this limited baseline awareness, most participants depended entirely on healthcare providers for initial education and device orientation.

### Information sources

Knowledge acquisition among participants was shaped by diverse sources, with healthcare providers serving as the primary source of information for all participants (*n* = 20). Provider-patient communication played a crucial role in facilitating device use, as participants often relied on their doctors or nurses to explain how to use the device and its purpose in managing their health. For example, one participant stated, “The person who brought it to me explained to me and showed me how to use it” (Interviewee 13).

In addition to healthcare providers, some participants supplemented their understanding through family members, friends, or self-directed learning. Four participants (20%) also received additional guidance from family or friends, and three (15%) relied on self-learning using manuals or written materials. One participant explained, “I think I just read the manual. I don’t think that there were any associated videos or anything like that” (Interviewee 5). However, the quality and depth of knowledge gained from these sources varied significantly. While some participants felt confident in their understanding, others expressed uncertainty, which often hindered consistent utilization of SHDs.

### Knowledge gaps

Knowledge gaps were a notable barrier to consistent SHDs use, as participants who lacked a clear understanding of the device’s functionality and purpose often reported uncertainty about its reliability. Additionally, knowledge gaps were also associated with negative outcomes, physical/mental barriers, and physical barriers (Table 4).

**Table 4:** Interactions between knowledge gaps and extraneous barriers identified across interviews (*N *= 12).

Interaction term	*N*	Subthemes and dimensions
Knowledge gaps × negative outcomes	5	No major lifestyle changes Reliance on doctor for interpretation Limited impact on doctor visits Missed monitoring reminders Reliance on healthcare team for updates
Knowledge gaps × psychological/mental barriers	4	Conflicting motivations Fear of hospitalization Fear of medical feedback Stress and mental load Work fatigue and time constraints
Knowledge gaps × physical barriers	1	Forgetfulness Need for assistance Pain and discomfort
Knowledge gaps × healthcare provider decision	2	Participants relied solely on healthcare providers for guidance

*Note*. **“***N*” represents the number of times each code was observed across participant interviews. A higher count reflects more frequent identification of the theme during the analysis.

Twelve participants (60%) had never heard of the SHD before being prescribed it and expressed uncertainty about SHD functionality. One participant reflected on their unfamiliarity with the technology: “I never had to use those before. No… Back in the days, they didn’t have these kinds of devices. So, this is something new. I didn’t know before. No” (Interviewee 16).

These gaps in knowledge were sometimes linked to inconsistent use patterns, with participants reporting discrepancies in how often they used the device compared to healthcare provider recommendations. The same participant quoted above initially used the device both morning and night, but later adjusted their routine based on personal experience rather than medical guidance:

I used to say morning and night, but when they stopped monitoring it, I used it every morning, but I didn’t use it at night unless I felt like I had to… I know when my blood pressure is too low because when it gets too low, it throws your body off… So, I just use it in the morning. But sometimes I would take it at night, even if I didn’t feel like it was too low, just to make sure it was OK to take my medicine. (Interviewee 16)

#### Negative outcomes

Of the 12 participants who reported knowledge gaps, 5 (42%) reported negative outcomes. One participant shared, “I could have somebody explain it, because I’m not computer-wise. I can’t say it changes my life because I wasn’t taking it every day” (Interviewee 7). This overlap underscores the compounded challenges faced by individuals unfamiliar with SHDs, as a lack of prior knowledge often hindered their ability to fully engage with the technology and integrate it effectively into their healthcare routines.

#### Psychological/mental barriers

Psychological and mental barriers were particularly prominent, with 4 of 12 (33%) participants expressing stress, anxiety, or conflicting feelings about using the device. For instance, one participant described their unease with external monitoring, stating, “I’d be a little freaked out if someone from the doctor’s office was calling me about my blood pressure reading. I mean, there’s an aspect of Big Brother here that’s not very appealing” (Interviewee 5). Another participant reported that work fatigue and irregular schedules often disrupted their ability to use the device consistently, saying, “I had started back to work, and with my work schedule, when I came in, I would just be tired and go to sleep and get up and go back to work” (Interviewee 8).

#### Physical barriers

Physical barriers were less common but still significant, with one (8%) participant reporting that arthritis occasionally made it difficult to use the device: “Sometimes my arm hurts. So that would be the only thing that would get in the way. My arm hurts sometimes, and I won’t use it” (Interviewee 10). Although they managed to use the device despite these challenges, their experience highlights the importance of designing SHDs that accommodate physical limitations. Despite these barriers, many participants became consistent users once gaps in knowledge were addressed through clear explanations and continued support.

### Facilitators of engagement

Some participants' use of the SHD was influenced by healthcare providers rather than personal choice. Two individuals reported relying entirely on their doctors’ guidance for operating and interpreting the device (Table 4). This reliance reflects the difficulties faced by individuals with lower digital knowledge, who may struggle to interpret device outputs independently and require additional assistance.

Despite these challenges, 18 (90%) participants reported regular use of the SHDs once they understood their purpose and were given clear guidance. Frequency and timing of use were often shaped by the knowledge imparted by healthcare providers, as illustrated by one participant who shared, “I check it every day when I can or wait for my caregiver to come and put it on my arm and take my blood pressure. It’s every day” (Interviewee 12).

### Behavioral impacts

Positive impacts of SHD use on health awareness, which lead to beneficial behavioral changes and better disease management, as well as improved emotional impacts, were observed among 17 participants (85%). Many participants highlighted how SHDs allowed them to monitor their health effectively and make informed choices. For instance, one participant explained, “It helped because I can see what’s going on. My diet. What makes it go well. What makes the level stay. So, it helps me make better choices of what I want to eat” (Interviewee 9). Another participant shared, “It lets me know what my blood pressure was when I took it. That’s how. It made me realize that the medication that they had prescribed for me is working. That’s actually in my life” (Interviewee 12).

This increased health awareness often led to behavioral changes. Participants described how SHDs prompted them to modify their diets, increase physical activity, and establish consistent health-monitoring routines. One participant noted, “I know if I eat this, it’s gonna run my blood pressure up. So, in order to keep it from not running up, I don’t eat it anymore. I stopped messing with it” (Interviewee 1). Another elaborated,

It helps me tremendously with my reading for my blood pressure. It helped me with my diet. It lets me know if I’m over or blood pressure is too high. It lets me know that I need to slow down. (Interviewee 14)

In addition to behavioral changes and better hypertension management, participants expressed that regular SHD use fostered emotional benefits, such as a greater sense of security and reduced anxiety about their health. As one participant explained, “It gives me some more security or confidence. Less worry about my blood pressure” (Interviewee 11). Others appreciated the independence and self-management that the SHD enabled. For example, one participant stated, “I am able to monitor my blood pressure, so that I can have some type of control over what’s going on and if I need to call the doctor or whatever. It’s very convenient” (Interviewee 19).

SHD also enhances participants’ interactions with healthcare providers by providing accurate data and fostering a proactive approach to health. One participant noted, “Using this blood pressure cuff has changed my experience seeing my doctor. It put me more in a positive mood of going to see him or talk to him on the phone” (Interviewee 4).

#### Clinically relevant patterns in SHD engagement

Patient engagement with SHDs was closely tied to both prior knowledge and provider involvement. Among the 12 participants (60%) unfamiliar with SHDs before prescription, many expressed confusion about device function and used it inconsistently unless given specific guidance. In contrast, after all 20 participants received clear instructions from healthcare providers and reported understanding how the device supported hypertension management, they were more likely to use it regularly (18 of 20, 90%). Participants frequently relied on providers to interpret device readings, highlighting the importance of clinical follow-up in SHD integration. Additionally, 85% of participants reported that using the SHD enhanced their awareness of blood pressure patterns and informed behavioral decisions, such as dietary changes or medication adherence. These patterns emphasize that structured education and follow-up may be critical for effective SHD use in routine hypertension care for racial-ethnic minority older adults.

## Discussion and implications

By integrating CRT with Andersen’s model, this study conceptualized knowledge as shaped not only by individual characteristics but also by structural conditions that influence access to information and capacity to act on it. This combined approach provides a more comprehensive understanding of disparities in digital health technology use among racial-ethnic minority older adults.

Findings indicate that knowledge and provider involvement are central to engagement with SHDs. Most participants had no prior awareness of SHDs before they were prescribed by healthcare providers. They also lacked an understanding of the purpose or use of SHDs until receiving instruction from the pharmacist in the larger study. The findings suggest that access to SHD-related knowledge is largely mediated through clinical encounters, positioning providers as critical gatekeepers of information. However, reliance on provider-mediated knowledge may inadvertently reinforce disparities. The older generation of racial-ethnic minority adults endured racial segregation and systemic inequities throughout the Civil Rights era. Historically, these communities have faced barriers to quality care and have been disproportionately exposed to unethical medical practices, such as the Tuskegee Syphilis Study. From a CRT perspective, historical and ongoing mistrust of healthcare systems among racial-ethnic minority populations may limit engagement with providers and, consequently, access to essential information (Ford & Airhihenbuwa, 2010; Freeman et al., 2017). Furthermore, previous studies indicated that some providers share less information and display less positive affect when treating racial-ethnic minority patients compared to White patients, contributing to lower levels of patient engagement and trust (Musa et al., 2009). As a result, disparities in knowledge acquisition may contribute to lower SHD utilization and, ultimately, continued inequalities in hypertension management and related health outcomes.

Knowledge gaps also emerged as a direct barrier to SHD use. Participants’ limited understanding of how devices function and their intended purpose contributed to their uncertainty, mistrust of both the technology and healthcare providers, and negative emotional responses, such as stress and anxiety. Consistent with prior research (Kim et al., 2023), these findings reinforce the digital literacy framework’s assertion that successful technology adoption requires both functional and conceptual knowledge (Oh et al., 2021; Vercruyssen et al., 2023). The identified subtheme of unfamiliarity with technology further reflects broader digital divide issues, including disparities in digital literacy (Oh et al., 2021; Vercruyssen et al., 2023). While some aspects of this divide may be generational (Győrffy et al., 2023), they are also shaped by structural inequalities that disproportionally affect racial-ethnic minority older adults, reinforcing barriers to engagement with digital tools.

These findings also align with technology adoption frameworks, including the Technology Acceptance Model (TAM) and the Unified Theory of Acceptance and Use of Technology (UTAUT). TAM proposes that perceived usefulness and ease of use influence willingness to adopt technology, and UTAUT highlights the importance of facilitating conditions and social influence in technology uptake (Ammenwerth Elske, 2019). The study showed that participants were more likely to adopt SHDs when healthcare providers explained the device’s purpose, provided instructions, and reinforced its role in hypertension management. In contrast, uncertainty about device functionality, limited digital familiarity, and concerns regarding monitoring reduced confidence and engagement. Healthcare providers’ recommendations and ongoing support were viewed as important facilitating conditions that increased participants’ willingness to use SHDs. However, our findings extend these models by demonstrating how structural inequities and historical mistrust shape knowledge and technology adoption among racial-ethnic minority older adults.

The role of healthcare providers as facilitators of SHD engagement is both promising and complex. Participants who trusted their providers were more likely to adopt SHDs and report positive behavioral health impacts. Improvements in behaviors and health using SHDs are well-documented (R. Li et al., 2025; Shan et al., 2020). However, this pathway assumes consistent access to and trust in healthcare providers—conditions that are not equally distributed. CRT helps contextualize these patterns by highlighting how structural racism shapes healthcare experiences, trust, and access. While provider engagement can support SHD adoption, reliance on this mechanism alone may perpetuate disparities if underlying structural barriers are not addressed.

### Implications for practice

Addressing the knowledge gap identified in this study may require thoughtful and targeted education strategies. Culturally responsive education designed specifically for racial-ethnic minority older adults may improve SHD engagement (Lambert et al., 2021). Educational interventions should focus not only on device operation but also on how SHD use contributes to blood pressure control and treatment decisions (Tappen et al., 2022; Yatabe et al., 2021). Community-based organizations and senior centers, which provide services and support to racial-ethnic minority older adults and already offer hypertension programs, may serve as valuable partners in delivering this content. Integrating SHD education into trusted, non-clinical settings could enhance digital literacy, reinforce self-monitoring behaviors, and improve chronic disease self-management among underserved populations (Yang et al., 2024).

Given the strong reliance participants placed on healthcare providers for SHD guidance, clinician engagement is a critical component of successful implementation. Similar findings from another study reported that a strong provider-patient relationship, provider engagement, and recommendations are pivotal in facilitating the adoption of digital health tools among racial-ethnic minority adults (Ordaz et al., 2021). Because trust is foundational, diversifying the medical workforce emerges as a critical strategy for improving knowledge of SHD use and promoting patient engagement. Prior studies suggested that racial concordance increases the willingness of racial-ethnic minority populations to voice concerns, seek advice, and participate in shared decision making (Takeshita et al., 2020). By receiving education on SHD functionality from trusted, racially concordant providers, racial-ethnic minority older adults are better equipped to adopt self-monitoring behaviors and improve chronic disease self-management. However, while increasing diversity is vital, it is also essential to foster cultural mindfulness and incorporate cultural competency training among all health professionals, ensuring equitable care is delivered to all populations.

Although diversifying health professionals could improve SHD use, racial-ethnic minority older adults often receive care in safety-net healthcare settings (SNHS), where providers face competing demands, limited staffing, and high burnout, reducing opportunities to provide and introduce SHDs (Welles et al., 2023). Moreover, SNHS frequently operate under financial and workforce constraints, limiting visit time, continuity of care, and access to digital health resources (Welles et al., 2023). These structural inequities may reduce patients’ exposure to SHDs and make SHD education challenging. While policy changes are necessary to improve funding and resource availability for SNHS, it will take several years to accomplish.

Scalable solutions, such as brief counseling scripts, one-page instructional handouts, or QR-linked video tutorials, could enable providers to reinforce SHD use efficiently during routine visits (Kokorelias et al., 2022). These tools could be integrated into hypertension workflows or electronic health record systems to ensure consistent messaging. Transitioning to a multidisciplinary, team-based care approach, including community health workers, social workers, pharmacists, and health educators, to deliver or reinforce SHD instruction improves racial-ethnic minority older adults’ understanding while minimizing workflow disruptions. Interacting with a diverse team allows racial-ethnic minority older adults to develop meaningful, trusted relationships with multiple members of the clinical team, increase confidence with SHD use, and expand their network of support. These strategies allow the clinical team to build on and diversify the existing trust between racial-ethnic minority older adults and their providers, an essential driver of SHD engagement observed in this study (Yatabe et al., 2021).

### Strengths and limitations

This study contributes new evidence on the role of SHDs in enhancing health engagement among racial-ethnic minority older adults, particularly through increased self-monitoring and patient-provider communication. While prior research has highlighted disparities in access and adoption of digital tools, our findings suggest that knowledge and guidance, not just technology access, are key barriers to equitable use (Tappen et al., 2022; Wang et al., 2021). These results are consistent with studies showing that digital tools can improve chronic disease self-management and strengthen clinical communication when properly supported (Yatabe et al., 2021). As digital health becomes increasingly central to chronic disease management, addressing these knowledge gaps is essential to avoid deepening existing health disparities. Future interventions should prioritize diversifying the medical workforce, culturally responsive education, accessible materials, and integration of SHD support into clinical and community-based care settings (Kokorelias et al., 2022).

While the findings highlight the importance of knowledge in utilizing SHDs, some methodological limitations temper their interpretation. First, it was conducted at a single academic medical center with a small, purposively sampled cohort of 20 participants, which limits generalizability. Second, the sample is largely homogeneous, with 95% identifying as Black American. It is possible that the findings regarding knowledge might be different for other racial-ethnic minority groups. Third, social desirability, recall bias, and reporting bias (Bispo Júnior, 2022) may have influenced the findings. Finally, participants in this study were enrolled in a larger study in which they received both a device and education on its use. This likely enhanced engagement and knowledge about SHDs. Future research should employ larger, multi-site studies with more diverse racial-ethnic minority populations and rigorous methods to mitigate these biases.

## Conclusion

SHD adoption supports blood pressure management and improves chronic disease outcomes, but limited knowledge remains a key barrier to its effective use among racial-ethnic minority older adults. This study highlights the importance of provider-delivered education, ongoing support, and culturally responsive communication in promoting SHD engagement. To reduce disparities in hypertension outcomes, clinical and public health strategies should prioritize scalable, culturally responsive education, recruit and train diverse health professionals, integrate SHD support into care delivery, and address digital literacy as essential to equitable chronic disease management.

## Data Availability

The data are not publicly available due to privacy concerns and confidentiality agreements. This study was not preregistered.

## References

[igag083-B1] Aaby A. , Friis K., Christensen B., Rowlands G., Maindal H. T. (2017). Health literacy is associated with health behaviour and self-reported health: A large population-based study in individuals with cardiovascular disease. European Journal of Preventive Cardiology, 24, 1880–1888. 10.1177/2047487317729538 28854822 PMC5680908

[igag083-B2] Aggarwal R. , Chiu N., Wadhera R. K., Moran A. E., Raber I., Shen C., Yeh R. W., Kazi D. S. (2021). Racial/ethnic disparities in hypertension prevalence, awareness, treatment, and control in the United States, 2013 to 2018. Hypertension, 78, 1719–1726. 10.1161/HYPERTENSIONAHA.121.17570 34365809 PMC10861176

[igag083-B3] Ammenwerth E. (2019). Technology acceptance models in health informatics: TAM and UTAUT. In Studies in health technology and informatics. IOS Press. 10.3233/SHTI190111 31411153

[igag083-B4] Anderson M. , Perrin A. (2017). *Tech adoption climbs among older Americans.* https://www.pewresearch.org/internet/2017/05/17/tech-adoption-climbs-among-older-adults/

[igag083-B5] Bazargan M. , Cobb S., Assari S. (2021). Discrimination and medical mistrust in a racially and ethnically diverse sample of California adults. The Annals of Family Medicine, 19, 4–15. 10.1370/afm.2632 33431385 PMC7800756

[igag083-B6] Bispo Júnior J. P. (2022). *Social desirability bias in qualitative health research.* 10.11606/s1518-8787.2022056004164 PMC9749714 36515303

[igag083-B7] Bradley E. H. , McGraw S. A., Curry L., Buckser A., King K. L., Kasl S. V., Andersen R. (2002). Expanding the Andersen model: The role of psychosocial factors in long‐term care use. Health Services Research, 37, 1221–1242. 10.1111/1475-6773.01053 12479494 PMC1464025

[igag083-B8] Centers for Disease Control and Prevention. (2023a, May 12). Estimated hypertension prevalence, treatment, and control among U.S. adults. Centers for Disease Control and Prevention. https://millionhearts.hhs.gov/data-reports/hypertension-prevalence.html

[igag083-B9] Centers for Disease Control and Prevention. (2023b, June). High Blood Pressure Facts. High Blood Pressure. https://www.cdc.gov/high-blood-pressure/data-research/facts-stats/index.html

[igag083-B10] Chandrasekaran R. , Katthula V., Moustakas E. (2021). *Too old for technology?* Use of wearable healthcare devices by older adults and their willingness to share health data with providers. Health Informatics Journal, 27, 14604582211058073. 10.1177/14604582211058073 34802315

[igag083-B11] Ciemins E. L. , Arora A., Coombs N. C., Holloway B., Mullette E. J., Garland R., Bishop-Green S., Penso J., Coon P. J. (2018). Improving blood pressure control using smart technology. Telemedicine and E-Health, 24, 222–228. 10.1089/tmj.2017.0028 28930497

[igag083-B12] Ford C. L. , Airhihenbuwa C. O. (2010). Critical race theory, race equity, and public health: Toward antiracism praxis. American Journal of Public Health, 100, S30–S35. 10.2105/AJPH.2009.171058 20147679 PMC2837428

[igag083-B13] Freeman R. , Gwadz M. V., Silverman E., Kutnick A., Leonard N. R., Ritchie A. S., Reed J., Martinez B. Y. (2017). Critical race theory as a tool for understanding poor engagement along the HIV care continuum among African American/Black and Hispanic persons living with HIV in the United States: A qualitative exploration. International Journal for Equity in Health, 16, 54. 10.1186/s12939-017-0549-3 28340589 PMC5364619

[igag083-B14] Glazier J. J. (2022). Pathophysiology, diagnosis, and management of hypertension in the elderly. International Journal of Angiology, 31, 222–228. 10.1055/s-0042-1759486 PMC9803548 36588864

[igag083-B15] Green J. , Thorogood N. (2018). Qualitative Methods for Health Research. SAGE Publications Ltd. 10.4135/9781036234386

[igag083-B16] Győrffy Z. , Boros J., Döbrössy B., Girasek E. (2023). Older adults in the digital health era: Insights on the digital health related knowledge, habits and attitudes of the 65 year and older population. BMC Geriatrics, 23, 779. 10.1186/s12877-023-04437-5 38012565 PMC10683351

[igag083-B17] Harmon Still C. , Jones L. M., Moss K. O., Variath M., Wright K. D. (2018). African American older adults’ perceived use of technology for hypertension self-management. Research in Gerontological Nursing, 11, 249–256. 10.3928/19404921-20180809-02 30230518 PMC6386647

[igag083-B18] Heaton J. , Alshami A., Imburgio S., Mararenko A., Schoenfeld M., Sealove B., Asif A., Almendral J. (2024). Current trends in hypertension identification and management: Insights from the National Health and Nutrition Examination Survey (NHANES) following the 2017 ACC/AHA high blood pressure guidelines. Journal of the American Heart Association, 13, e034322. 10.1161/JAHA.123.034322 38563377 PMC11262485

[igag083-B19] Hellem A. , Whitfield C., Mansour M., Curran Y., Dinh M., Warden K., Skolarus L. E. (2024). Determinants of bluetooth-enabled self-measured blood pressure monitoring in federally qualified health centers. Journal of Primary Care & Community Health, 15, 21501319241229921. 10.1177/21501319241229921 PMC10894531 38400549

[igag083-B20] Jo S. , Kim S., Park K., Kim H., Han S., Park W. (2019). Self‐blood pressure monitoring is associated with improved awareness, adherence, and attainment of target blood pressure goals: Prospective observational study of 7751 patients. The Journal of Clinical Hypertension, 21, 1298–1304. 10.1111/jch.13647 31393062 PMC8030418

[igag083-B21] Kaur K. , Abu-Qamar M. Z., Rashidi A., McKay N., Saunders R. (2025). Applying hybrid coding and reflexivity in qualitative content analysis: An exemplar. Global Qualitative Nursing Research, 12, 23333936251395024. 10.1177/23333936251395024 41283066 PMC12638670

[igag083-B22] Kim H.-N. , Freddolino P. P., Greenhow C. (2023). Older adults’ technology anxiety as a barrier to digital inclusion: A scoping review. Educational Gerontology, 49, 1021–1038. 10.1080/03601277.2023.2202080

[igag083-B23] Kokorelias K. M. , Nelson M. L., Tang T., Steele Gray C., Ellen M., Plett D., Jarach C. M., Xin Nie J., Thavorn K., Singh H. (2022). Inclusion of older adults in digital health technologies to support hospital-to-home transitions: Secondary analysis of a rapid review and equity-informed recommendations. JMIR Aging, 5, e35925. 10.2196/35925 35475971 PMC9096639

[igag083-B24] Kulkarni A. , Mehta A., Yang E., Parapid Biljana. (n.d.). Older adults and hypertension: Beyond the 2017 guideline for prevention, detection, evaluation, and management of high blood pressure in adults. American College of Cardiology. Retrieved February 18, 2025. https://www.acc.org/latest-in-cardiology/articles/2020/02/26/06/24/older-adults-and-hypertension

[igag083-B25] Ladin K. , Porteny T., Perugini J. M., Gonzales K. M., Aufort K. E., Levine S. K., Wong J. B., Isakova T., Rifkin D., Gordon E. J., Rossi A., Koch-Weser S., Weiner D. E. (2021). Perceptions of telehealth vs in-person visits among older adults with advanced kidney disease, care partners, and clinicians. JAMA Network Open, 4, e2137193. 10.1001/jamanetworkopen.2021.37193 34870680 PMC8649833

[igag083-B26] Lambert S. , Schaffler J. L., Ould Brahim L., Belzile E., Laizner A. M., Folch N., Rosenberg E., Maheu C., Ciofani L., Dubois S., Gélinas-Phaneuf E., Drouin S., Leung K., Tremblay S., Clayberg K., Ciampi A. (2021). The effect of culturally-adapted health education interventions among culturally and linguistically diverse (CALD) patients with a chronic illness: A meta-analysis and descriptive systematic review. Patient Education and Counseling, 104, 1608–1635. 10.1016/j.pec.2021.01.023 33573916

[igag083-B27] Li R. , Li Y., Wang L., Li L., Fu C., Hu D., Wei Q. (2025). Wearable activity tracker–based interventions for physical activity, body composition, and physical function among community-dwelling older adults: Systematic review and meta-analysis of randomized controlled trials. Journal of Medical Internet Research, 27, e59507. 10.2196/59507 40179387 PMC12006780

[igag083-B28] Li Y. , Spoer B. R., Lampe T. M., Hsieh P. Y., Nelson I. S., Vierse A., Thorpe L. E., Gourevitch M. N. (2023). Racial/ethnic and income disparities in neighborhood-level broadband access in 905 US cities, 2017–2021. Public Health, 217, 205–211. 10.1016/j.puhe.2023.02.001 36917875 PMC10688393

[igag083-B29] Malterud K. , Siersma V. D., Guassora A. D. (2016). Sample size in qualitative interview studies: Guided by information power. Qualitative Health Research, 26, 1753–1760. 10.1177/1049732315617444 26613970

[igag083-B30] Muntner P. , Hardy S. T., Fine L. J., Jaeger B. C., Wozniak G., Levitan E. B., Colantonio L. D. (2020). Trends in blood pressure control among US adults with hypertension, 1999-2000 to 2017-2018. JAMA, 324, 1190. 10.1001/jama.2020.14545 32902588 PMC7489367

[igag083-B31] Musa D. , Schulz R., Harris R., Silverman M., Thomas S. B. (2009). Trust in the health care system and the use of preventive health services by older Black and White adults. American Journal of Public Health, 99, 1293–1299. 10.2105/AJPH.2007.123927 18923129 PMC2696665

[igag083-B32] Naeem M. , Ozuem W., Howell K., Ranfagni S. (2023). A step-by-step process of thematic analysis to develop a conceptual model in qualitative research. International Journal of Qualitative Methods, 22, 16094069231205789. 10.1177/16094069231205789

[igag083-B33] Norman C. D. , Skinner H. A. (2006). eHealth literacy: Essential skills for consumer health in a networked world. Journal of Medical Internet Research, 8, e9. 10.2196/jmir.8.2.e9 16867972 PMC1550701

[igag083-B34] Nowell L. S. , Norris J. M., White D. E., Moules N. J. (2017). Thematic analysis: Striving to meet the trustworthiness criteria. International Journal of Qualitative Methods, 16, 1–13. 10.1177/1609406917733847

[igag083-B35] Oh S. S. , Kim K.-A., Kim M., Oh J., Chu S. H., Choi J. (2021). Measurement of digital literacy among older adults: Systematic review. Journal of Medical Internet Research, 23, e26145. 10.2196/26145 33533727 PMC7889415

[igag083-B36] Ordaz O. H. , Croff R. L., Robinson L. D., Shea S. A., Bowles N. P. (2021). Optimization of primary care among Black Americans using patient portals: Qualitative study. Journal of Medical Internet Research, 23, e27820. 10.2196/27820 34081016 PMC8212618

[igag083-B37] Rodriguez-Elliott S. , Vachuska K. (2023). Measuring the digital divide: A neighborhood-level analysis of racial inequality in internet speed during the COVID-19 pandemic. Societies, 13, 92. 10.3390/soc13040092

[igag083-B38] Shan R. , Ding J., Weng D., Spaulding E. M., Wongvibulsin S., Lee M. A., Demo R., Marvel F. A., Martin S. S. (2020). Blood pressure control over 30 days after acute myocardial infarction: Insights from the Corrie health digital platform. American Journal of Preventive Cardiology, 3, 100054. 10.1016/j.ajpc.2020.100054 PMC7497394 32964212

[igag083-B39] Stergiou G. S. , Kario K., Kollias A., McManus R. J., Ohkubo T., Parati G., Imai Y. (2018). Home blood pressure monitoring in the 21st century. The Journal of Clinical Hypertension, 20, 1116–1121. 10.1111/jch.13284 30003694 PMC8030885

[igag083-B40] Takeshita J. , Wang S., Loren A. W., Mitra N., Shults J., Shin D. B., Sawinski D. L. (2020). Association of racial/ethnic and gender concordance between patients and physicians with patient experience ratings. JAMA Network Open, 3, e2024583. 10.1001/jamanetworkopen.2020.24583 33165609 PMC7653497

[igag083-B41] Tappen R. M. , Cooley M. E., Luckmann R., Panday S. (2022). Digital health information disparities in older adults: A mixed methods study. Journal of Racial and Ethnic Health Disparities, 9, 82–92. 10.1007/s40615-020-00931-3 33415705 PMC7790471

[igag083-B42] Tong A. , Sainsbury P., Craig J. (2007). Consolidated criteria for reporting qualitative research (COREQ): A 32-item checklist for interviews and focus groups. International Journal for Quality in Health Care, 19, 349–357. 10.1093/intqhc/mzm042 17872937

[igag083-B43] Travers J. L. , Hirschman K. B., Naylor M. D. (2020). Adapting Andersen’s expanded behavioral model of health services use to include older adults receiving long-term services and supports. BMC Geriatrics, 20, 58. 10.1186/s12877-019-1405-7 32059643 PMC7023712

[igag083-B44] Vercruyssen A. , Schirmer W., Geerts N., Mortelmans D. (2023). How “basic” is basic digital literacy for older adults? Insights from digital skills instructors. Frontiers in Education, 8, 1231701. 10.3389/feduc.2023.1231701

[igag083-B45] Wang J. , Li Y., Chia Y., Cheng H., Minh H. V., Siddique S., Sogunuru G. P., Tay J. C., Teo B. W., Tsoi K., Turana Y., Wang T., Zhang Y., Kario K., & the Hypertension Cardiovascular Outcome Prevention, Evidence (HOPE) Asia Network. (2021). Telemedicine in the management of hypertension: Evolving technological platforms for blood pressure telemonitoring. The Journal of Clinical Hypertension, 23, 435–439. 10.1111/jch.14194 33484617 PMC8029526

[igag083-B46] Welles C. C. , Tong A., Brereton E., Steiner J. F., Wynia M. K., Powe N. R., Chonchol M., Hasnain-Wynia R., Cervantes L. (2023). Sources of clinician burnout in providing care for underserved patients in a safety-net healthcare system. Journal of General Internal Medicine, 38, 1468–1475. 10.1007/s11606-022-07896-5 36376633 PMC10160271

[igag083-B47] Yang R. , Gao S., Jiang Y. (2024). Digital divide as a determinant of health in the U.S. older adults: Prevalence, trends, and risk factors. BMC Geriatrics, 24, 1027. 10.1186/s12877-024-05612-y 39709341 PMC11662839

[igag083-B48] Yatabe J. , Yatabe M. S., Okada R., Ichihara A. (2021). Efficacy of telemedicine in hypertension care through home blood pressure monitoring and videoconferencing: Randomized controlled trial. JMIR Cardio, 5, e27347. 10.2196/27347 34321194 PMC8441608

